# Multidimensional visualization and analysis of chronic pain variables of patients who attended a chronic pain program

**DOI:** 10.3389/fpain.2023.1125992

**Published:** 2023-10-24

**Authors:** Yuelin (Cindy) Li, Eleni G. Hapidou

**Affiliations:** ^1^ University of London Worldwide, London, United Kingdom; ^2^ Michael G. DeGroote Pain Clinic, Hamilton Health Sciences, Hamilton, ON, Canada; ^3^ McMaster University, Hamilton, ON, Canada

**Keywords:** chronic pain, visualization, biplot, canonical correlation analyses (CCA), outcomes—health care

## Abstract

Psychologically-based chronic pain variables measure multiple domains of the pain experience such as anxiety, depression, catastrophizing, acceptance and stages of change. These variables measure specific areas such as emotional and cognitive states towards chronic pain and its management, acceptance towards the chronic pain condition, and an individual’s readiness to move towards self-management methods. Conceptually, these variables appear to be interrelated to each other, and also form groupings of similar underlying themes. Groupings that have been previously discussed for these variables include positive and negative affect, and improved and poor adjustment. Psychological experience of chronic pain as a whole is mostly understood through conceptually consolidating individual scores across different measures covering multiple domains. A map of these variables in relation to each other can offer an overview for further understanding and exploration. We hereby visualize highlights of relationships among 11 psychosocial chronic pain variables including measures examining physical and somatic aspects, using three-dimensional biplots. Variables roughly form two groupings, with one grouping consisting of items of negative affect, cognition, and physical state ratings, and the other grouping consisting of items of acceptance and the later three stages of change (contemplation, action, maintenance). Also, we follow up with canonical correlation as a complement to further identify key relationships between bimodal groupings. Key variables linking bimodal relationships consist of catastrophizing, depression and anxiety in one grouping and activity engagement in the other. Results are discussed in the context of existing literature.

## Introduction

The biopsychosocial model conceptualizes pain from three perspectives, the physiological, psychological and sociocultural and their interactions (1). The psychological portion of the model further comprises individual factors such as affect, cognition, acceptance and stages of change. Affective factors include anxiety, depression, and catastrophizing, which are negative in quality (2). Multiple anxiety and trauma-related disorders such as panic and posttraumatic stress (PTSD) are associated with chronic pain and pain-related impairment (3, 4). The presence of pain or depression negatively influences treatment outcomes of the other, and these two factors may also interact with each other to negatively influence pain outcomes (5). Anxiety, together with depression increases odds of disabling pain, even with anxiety or depression in remission (6).

Anxiety and depression can interact with cognitive factors such as catastrophizing, which is a mediator for both anxiety and depression, and has also been found to predict depressive symptoms (7–9). Conversely, anxiety and depression can potentiate catastrophizing (10).

In addition to affective factors, chronic pain experiences have been captured through variables with positive qualities including pain acceptance and stages of change. These are related to improved functioning (11), and are intertwined with negative affect and cognition. The pain willingness (PW) and activities engagement (AE) subscales of the Chronic Pain Acceptance Questionnaire (CPAQ) are negatively associated with depressive symptoms and pain intensity and positively associated with functioning and work (12, 13). Acceptance is a mediator for the effect of pain on catastrophizing (14). In a study that examined the effects of a multidisciplinary CBT-based intervention on pain intensity and interference, only catastrophizing and AE were significant predictors of changes in pain and interference (15). Catastrophizing also has a notable influence on the relationship between acceptance and chronic pain adjustment (16).

The Pain Stages of Change Questionnaire (PSOCQ) is also intermingled with pain affect. Patients with chronic pain have different beliefs regarding pain management, and changing behaviour might be related to changes in stages of readiness (17, 18). Anxiety and depression significantly predicted the pre-contemplation subscale of the PSOCQ (19) in patients with chronic pain assessed at multidisciplinary pain clinics. Lower depression scores were associated with decreased scores in pre-contemplation and increased scores in maintenance pre- to post-treatment from interdisciplinary pain management programs (20, 21). Also, reductions in pre-contemplation and increases in action/maintenance were associated with improvements in functioning and pain (20). Change scores in pre-contemplation before and after pain neuroscience education were positively related to change scores in pain catastrophizing (22). In patients with fibromyalgia, increase in contemplation was associated with increase in catastrophizing, and post-treatment, depression was associated with contemplation in pain clinic patients (23). For patients in the action/maintenance stages, higher scores on weekly positive affect were associated with lower weekly pain perception (24).

These different measures make up two groups of factors: one consisting of negative affect/poor adjustment to chronic pain (depression, anxiety, catastrophizing), and the other consisting of positive affect/improved adjustment to chronic pain (CPAQ, PSOCQ) (11). Pain management aims at decreasing negative affect/poor adjustment and increasing positive affect/improved adjustment. These two groups may be bivalent, consisting of negative affect/poor adjustment and positive affect/improved adjustment (25). However, according to the Dynamic Affect Model, the differentiation between positive and negative affect is not as clear during stressful events such as pain and this may show variation between people as well. In chronic pain, which is a stressful event, there is more overlap between positive and negative affect (26). This study aims to explore the relationship between positive and negative factors, how these conceptual groups are reflected in practice, and which factors are key in the connection between the positive and negative.

## Methods

Data were collected as described in Li and Hapidou (27). In short, participants were adults attending a four-week chronic pain management program from 2007 to 2017. The program has been described in Williams et al. (21). The University and Hospital Ethics Board reviewed the study protocol and determined ethical approval for retrospective data analysis was not necessary.

### Brief description of the sample

The average age (SD) of the participants was 44.20 (10.28), with 49% males and 51% females. Their educational level was 13.35 years (3.24) and their pain duration was 64.99 (76.20). The majority, 80%, were Canadian-born and 65% were employed. The majority of patients had musculoskeletal pain.

Previous data analysis demonstrated highly significant changes between admission and discharge (*p* < 0.001) on the variables administered in the program such as pain intensity, depression, catastrophizing, anxiety, stages of change and pain acceptance (27).

### Data analysis

Principal component analysis (PCA) examined the structure and relationship of psychometric variables at admission and discharge (28). Then, the principal components were scaled and plotted on three-dimensional variable biplots for visualization. Biplots allow visual representation of the relationships among variables. A variable biplot focuses on relationships among variables (29).

The angle between the variable vectors represents correlation between the variables, and the length of the vector represents the variance of the variable (30).

A three-dimensional biplot includes the first 3 eigenvectors, and can capture the dimensions to a greater degree as compared to a two-dimensional representation (31).

The lengths of the vectors in the biplot were calculated as follows: the first 3 principal components in the admission and discharge datasets were scaled by the square root of their respective eigenvalues (32).

Canonical correlation analysis was conducted on both admission and discharge datasets, dividing the variables into two conceptual sets: negative affect/poor adjustment, positive affect/improved adjustment (33, 34). Canonical correlation can be used as a complement to PCA to further examine and concur structural relations (35).

Statistical analysis was conducted using R Statistical Software (v4.1.1) and RStudio (v2021.09.0.351) (36, 37). Biplots were created using the plotly package (v4.10.0) (38). Kaiser–Meyer–Olkin test and Bartlett’s test of sphericity were conducted using the psych package (v2.1.9) (39). Canonical correlation analysis was conducted using the CCA package (v1.2.1) and significance tests were conducted using the CCP package (v1.1) (40, 41).

## Results

Principal component analysis (PCA) was performed on admission and discharge datasets from a total of 927 patients with chronic pain who attended a four-week interdisciplinary chronic pain management program.

There were complete data on the 11 variables from 780 patients in the admission dataset, and from 797 patients in the discharge dataset.

Outliers were calculated with Mahalanobis distances, with criterion at *p* < 0.001 (28). After removing outliers, there were 770 patients in the admission dataset and 788 patients in the discharge dataset.

Variables were overall normally distributed as assessed by their graphed distribution (28).

Assumptions of linearity between 2 variables within the admission and discharge set were also met, as assessed by bivariate scatterplots (28).

The large samples were adequate for analysis. The Kaiser-Meyer-Olkin test was 0.83 for the admission dataset, and 0.89 for the discharge dataset (42).

The determinant of the correlation matrices for admission and discharge were non-zero and there were enough correlations between the variables to conduct dimensional analysis (43). Bartlett’s test of sphericity for the admission dataset was 3120.79 (*p* < 0.001) and 4,330.22 (*p* < 0.001) for the discharge dataset (44).

There were two moderately high correlations, between the action and maintenance subscales of the PSOCQ (correlation = 0.73 for admission, correlation = 0.75 for discharge) (See Tables 1, 2). There were no correlations above 0.90. For the purposes of structural and dimensional analysis, presence of higher correlations will not affect the strength of the analysis (28).

**Table 1:** Correlation table of variables in admission dataset.

Admission	1	2	3	4	5	6	7	8	9	10
1. Pain Intensity	1									
2. Depression	0.29***	1								
3. Catastrophizing	0.33***	0.64***	1							
4. Anxiety	0.26***	0.72***	0.58***	1						
5. Somatic Symptoms	0.21***	0.59***	0.48***	0.57***	1					
6. Activity Engagement	−0.26***	−0.50***	−0.49***	−0.44***	−0.33***	1				
7. Pain Willingness	−0.23***	−0.35***	−0.47***	−0.30***	−0.27***	0.33***	1			
8. Pre-contemplation	0.21***	0.33***	0.45***	0.25***	0.22***	−0.29***	−0.34***	1		
9. Contemplation	−0.02	−0.05	−0.05	−0.04	0.05	0.08*	−0.15***	−0.21***	1	
10. Action	−0.09**	−0.24***	−0.28***	−0.16***	−0.10**	0.36***	0.09*	−0.30***	0.25***	1
11. Maintenance	−0.13***	−0.28***	−0.31***	−0.22***	−0.15***	0.43***	0.11***	−0.24***	0.16***	0.73***

Pain Intensity = Pain Intensity Scale; Depression = Center for Epidemiological Studies-Depressed Mood Scale; Catastrophizing = Pain Catastrophizing Scale; Anxiety = Clinical Anxiety Scale; Somatic Symptoms = Patient Questionnaire of the PRIME-MD; Activity Engagement = Chronic Pain Acceptance Questionnaire – Activity Engagement; Pain Willingness = Chronic Pain Acceptance Questionnaire – Pain Willingness; Pre-contemplation = Pain Stages of Change Questionnaire – Pre-contemplation; Contemplation = Pain Stages of Change Questionnaire – Contemplation; Action = Pain Stages of Change Questionnaire – Action; Maintenance = Pain Stages of Change Questionnaire – Maintenance.

* <0.05.

** *p* < 0.01.

*** *p* < 0.001.

**Table 2:** Correlation table of variables in discharge dataset.

Discharge	1	2	3	4	5	6	7	8	9	10
1. Pain Intensity	1									
2. Depression	0.35***	1								
3. Catastrophizing	0.34***	0.66***	1							
4. Anxiety	0.31***	0.74***	0.65***	1						
5. Somatic Symptoms	0.29***	0.65***	0.53***	0.64***	1					
6. Activity Engagement	−0.28***	−0.55***	−0.56***	−0.48***	−0.43***	1				
7. Pain Willingness	−0.18***	−0.33***	−0.54***	−0.33***	−0.26***	0.27***	1			
8. Pre-contemplation	0.26***	0.43***	0.56***	0.36***	0.33***	−0.45***	−0.30***	1		
9. Contemplation	−0.20***	−0.21***	−0.20***	−0.16***	−0.13***	0.30***	−0.06	−0.41***	1	
10. Action	−0.32***	−0.40***	−0.39***	−0.30***	−0.29***	0.46***	0.08*	−0.53***	0.59***	1
11. Maintenance	−0.30***	−0.44***	−0.39***	−0.33***	−0.31***	0.48***	0.08*	−0.54***	0.56***	0.75***

Pain Intensity = Pain Intensity Scale; Depression = Center for Epidemiological Studies-Depressed Mood Scale; Catastrophizing = Pain Catastrophizing Scale; Anxiety = Clinical Anxiety Scale; Somatic Symptoms = Patient Questionnaire of the PRIME-MD; Activity Engagement = Chronic Pain Acceptance Questionnaire – Activity Engagement; Pain Willingness = Chronic Pain Acceptance Questionnaire – Pain Willingness; Pre-contemplation = Pain Stages of Change Questionnaire – Pre-contemplation; Contemplation = Pain Stages of Change Questionnaire – Contemplation; Action = Pain Stages of Change Questionnaire – Action; Maintenance = Pain Stages of Change Questionnaire – Maintenance.

* <0.05.

** *p* < 0.01.

*** *p* < 0.005.

**Figure 1:**
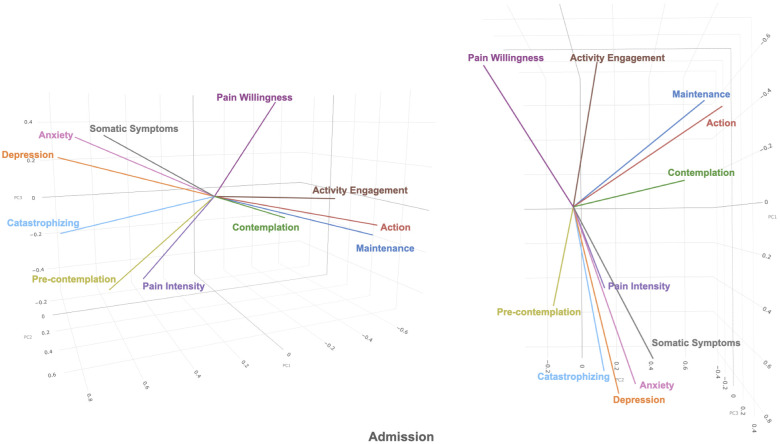
3-dimensional biplot of principal component 1, 2, and 3 (shown as PC1, PC2, PC3 in figure) of admission dataset.

**Figure 2:**
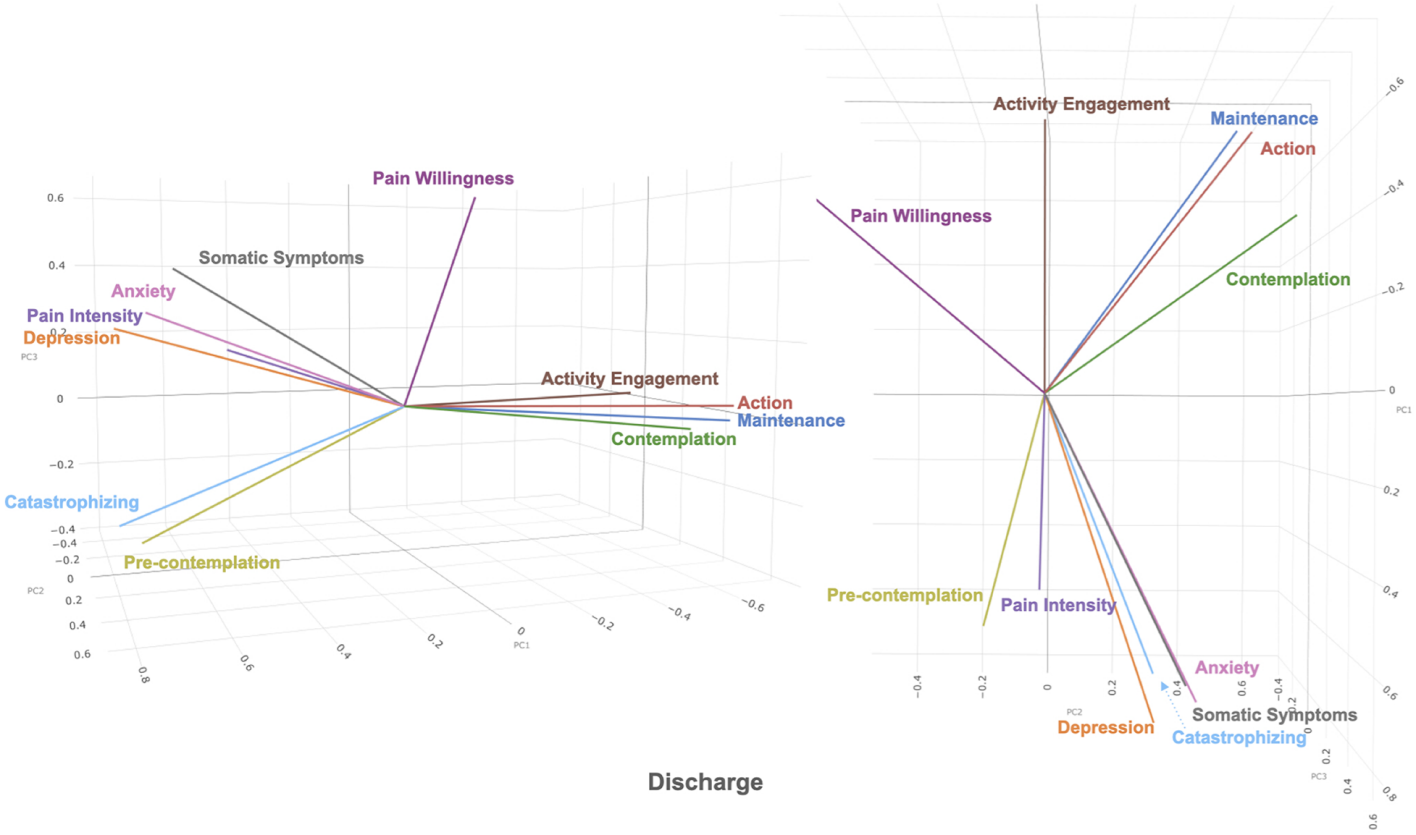
3-dimensional biplot of principal component 1, 2, and 3 (shown as PC1, PC2, PC3 in figure) of discharge dataset.

The first 3 principal components in the admission dataset and the discharge dataset were plotted in 3-dimensional biplots (See Figures 1, 2). Principal components and eigenvalues are shown in Tables 3, 4. Admission and discharge structures were similar, with roughly two groupings. One grouping consisted of somatic symptoms, anxiety, depression, catastrophizing, pain intensity and pre-contemplation. The other grouping consisted of activity engagement, action, maintenance, and contemplation. The vector for pain willingness leaned towards the second grouping. Vectors within the groupings became more tightly correlated at discharge as compared to admission, as shown by the distance between their vectors in the biplot.

**Table 3:** Principal components of admission and discharge datasets. Principal components consist of eigenvectors scaled by the square root of eigenvalues.

Admission	Variable	PC1	PC2	PC3	PC4	PC5	PC6	PC7	PC8	PC9	PC10	PC11
	Pain Intensity	0.44	−0.14	0.37	−0.12	−0.79	0.12	−0.03	0.01	−0.03	0.00	−0.01
	Depression	0.82	−0.21	−0.25	−0.09	0.02	−0.01	0.01	−0.18	−0.10	0.38	0.16
	Catastrophizing	0.82	−0.14	0.10	−0.04	0.09	0.00	0.00	−0.22	0.49	−0.07	−0.02
	Anxiety	0.75	−0.28	−0.34	−0.14	0.00	−0.04	0.00	−0.26	−0.25	−0.27	−0.13
	Somatic Symptoms	0.64	−0.36	−0.35	−0.08	0.06	0.30	−0.12	0.47	0.07	−0.03	−0.01
	Activity Engagement	−0.71	−0.12	0.04	−0.18	0.09	0.52	−0.32	−0.25	−0.01	−0.01	0.05
	Pain Willingness	−0.54	0.30	−0.52	−0.26	−0.25	0.20	0.41	−0.06	0.12	0.00	0.00
	Pre-contemplation	0.56	0.16	0.51	−0.29	0.32	0.28	0.35	0.04	−0.13	−0.03	0.03
	Contemplation	−0.13	−0.54	0.03	0.75	−0.02	0.24	0.25	−0.07	−0.02	0.01	−0.03
	Action	−0.49	−0.73	0.05	−0.23	0.00	−0.18	0.11	0.03	0.02	−0.14	0.32
	Maintenance	−0.53	−0.66	0.12	−0.35	0.08	−0.10	0.07	0.02	0.03	0.17	−0.31
Discharge	Variable	PC1	PC2	PC3	PC4	PC5	PC6	PC7	PC8	PC9	PC10	PC11
	Pain Intensity	0.50	−0.01	0.17	−0.85	−0.03	0.05	−0.07	0.02	−0.02	−0.01	0.00
	Depression	0.81	0.28	0.24	0.10	0.04	−0.01	0.01	−0.19	−0.23	0.32	−0.08
	Catastrophizing	0.81	0.31	−0.22	0.02	0.03	0.02	−0.12	−0.13	0.32	0.00	−0.26
	Anxiety	0.75	0.38	0.28	0.11	0.11	−0.12	−0.09	−0.25	0.01	−0.24	0.23
	Somatic Symptoms	0.68	0.34	0.38	0.12	0.17	0.06	0.14	0.45	0.05	−0.02	−0.03
	Activity Engagement	−0.73	0.00	0.01	−0.13	0.63	0.01	0.16	−0.13	0.03	0.02	−0.05
	Pain Willingness	−0.43	−0.50	0.64	0.12	−0.04	0.28	−0.20	−0.10	0.12	0.01	−0.07
	Pre-contemplation	0.71	−0.19	−0.38	0.08	0.21	0.44	−0.22	0.06	−0.10	−0.01	0.11
	Contemplation	−0.48	0.67	0.00	−0.04	−0.20	0.41	0.30	−0.12	0.04	−0.01	0.04
	Action	−0.69	0.55	0.03	0.00	0.02	0.01	−0.29	0.05	−0.24	−0.17	−0.22
	Maintenance	−0.71	0.51	−0.01	−0.04	0.04	−0.06	−0.32	0.10	0.16	0.23	0.20

Pain Intensity = Pain Intensity Scale; Depression = Center for Epidemiological Studies-Depressed Mood Scale; Catastrophizing = Pain Catastrophizing Scale; Anxiety = Clinical Anxiety Scale; Somatic Symptoms = Patient Questionnaire of the PRIME-MD; Activity Engagement = Chronic Pain Acceptance Questionnaire – Activity Engagement; Pain Willingness = Chronic Pain Acceptance Questionnaire – Pain Willingness; Pre-contemplation = Pain Stages of Change Questionnaire – Pre-contemplation; Contemplation = Pain Stages of Change Questionnaire – Contemplation; Action = Pain Stages of Change Questionnaire – Action; Maintenance = Pain Stages of Change Questionnaire – Maintenance.

**Table 4:** Eigenvalues of principal component analysis of admission and discharge datasets.

Admission	Principal Component	Eigenvalue	Proportion Variance	Cumulative % Variance
	1	4.14	37.64%	37.64%
	2	1.68	15.25%	52.89%
	3	0.99	9.02%	61.91%
	4	0.97	8.85%	70.76%
	5	0.82	7.42%	78.18%
	6	0.60	5.44%	83.62%
	7	0.48	4.41%	88.02%
	8	0.44	4.02%	92.05%
	9	0.35	3.19%	95.24%
	10	0.27	2.49%	97.73%
	11	0.25	2.27%	100.00%
Discharge	Principal Component	Eigenvalue	Proportion Variance	Cumulative % Variance
	1	5.00	45.47%	45.47%
	2	1.74	15.81%	61.28%
	3	0.90	8.19%	69.47%
	4	0.80	7.25%	76.72%
	5	0.53	4.86%	81.58%
	6	0.47	4.23%	85.81%
	7	0.43	3.91%	89.72%
	8	0.38	3.48%	93.20%
	9	0.27	2.43%	95.63%
	10	0.24	2.22%	97.85%
	11	0.24	2.15%	100.00%

Canonical correlation was performed between the two sets of variables representing negative affect/poor adjustment (somatic symptoms, anxiety, depression, catastrophizing, pain intensity) and positive affect/ improved adjustment (CPAQ, PSOCQ). For admission, the first canonical correlation was 0.68, the second was 0.20, the third was 0.12, and the following two correlations were close to zero. The F-test was F (30, 3080.00) = 18.65, *p* < 0.001 for all 5 canonical correlations. After removing the first canonical correlation, the F-test value was F (20, 2521.59) = 2.41, *p* < 0.001. Removing the second canonical correlation and subsequent removals produced F-tests that were not significant. Therefore, significant relationships between the two groupings were captured by the first two pairs of canonical variates.

Table 5 shows standardized canonical variate coefficients, correlations between the variates and canonical variates, proportion of variance, redundancy and canonical correlations for the admission data. From total proportion variance and redundancy, the first pair of canonical variates were moderately related, and the second pair of canonical variates were weakly related.

**Table 5:** Canonical correlation analysis of the admission dataset. Variables grouped by negative affect/ poor adjustment (Pain Intensity, Depression, Catastrophizing, Anxiety, Somatic Symptoms) and positive affect/ improved adjustment (Activity Engagement, Pain Willingness, Pre-contemplation, Contemplation, Action, Maintenance).

Admission	First Canonical Variate		Second Canonical Variate	
	Correlation	Function Coefficient	Correlation	Function Coefficient
Negative Affect/ Poor Adjustment Set Variables				
Pain Intensity	0.47	0.15	0.07	0.06
Depression	0.82	0.32	0.43	0.49
Catastrophizing	0.95	0.69	−0.24	−1.05
Anxiety	0.69	0.03	0.61	0.91
Somatic Symptoms	0.55	−0.01	0.22	−0.09
Proportion of Variance	0.52		0.13	Total = 0.65
Redundancy	0.24		0.01	Total = 0.25
Positive Affect/ Improved Adjustment Set Variables				
Activity Engagement	−0.81	−0.52	−0.56	−0.95
Pain Willingness	−0.71	−0.40	0.32	0.41
Pre-contemplation	0.67	0.35	−0.47	−0.53
Contemplation	−0.08	0.00	−0.07	−0.13
Action	−0.42	−0.01	0.18	0.42
Maintenance	−0.48	−0.12	−0.03	−0.08
Proportion of Variance	0.34		0.11	Total = 0.45
Redundancy	0.15		0.00	Total = 0.15
Canonical correlation	0.68		0.20	
Canonical correlation squared	0.46		0.04	

Pain Intensity = Pain Intensity Scale; Depression = Center for Epidemiological Studies-Depressed Mood Scale; Catastrophizing = Pain Catastrophizing Scale; Anxiety = Clinical Anxiety Scale; Somatic Symptoms = Patient Questionnaire of the PRIME-MD; Activity Engagement = Chronic Pain Acceptance Questionnaire – Activity Engagement; Pain Willingness = Chronic Pain Acceptance Questionnaire – Pain Willingness; Pre-contemplation = Pain Stages of Change Questionnaire – Pre-contemplation; Contemplation = Pain Stages of Change Questionnaire – Contemplation; Action = Pain Stages of Change Questionnaire – Action; Maintenance = Pain Stages of Change Questionnaire – Maintenance.

All variables in the negative affect/poor adjustment set were correlated with the first canonical variate, with all correlations of these variables above 0.3 (28). The strongest correlations were catastrophizing (0.95) and depression (0.82). Except for contemplation, all variables in the positive affect/ improved adjustment set were correlated with the first canonical variable. The strongest correlation was activity engagement (−0.81). The first canonical variate indicated that increases in catastrophizing, depression, pain intensity, anxiety, and somatic symptoms were associated with decreases in activity engagement, pain willingness, action and maintenance and increase in pre-contemplation.

The second canonical variate consisted of depression (0.43) and anxiety (0.61) in the negative affect/poor adjustment set, and activity engagement (−0.56) and pre-contemplation (−0.47) in the positive affect/ improved adjustment set. The second canonical variate showed that decreased depression and anxiety were associated with increase in activity engagement, and pre-contemplation.

For discharge, the first canonical correlation was 0.76, the second was 0.30, the third was 0.16 and the following two correlations were close to zero. The F-test was F (30, 3110.00) = 29.05, *p* < 0.001 for all 5 canonical correlations. After removing the first canonical correlation, the F-test value was F (20, 2581.28) = 4.96, *p* < 0.001. Removing the third canonical correlation and subsequent removals produced F-tests that were not significant (*p* > 0.001). Therefore, significant relationships between the two groupings were captured by the first two pairs of canonical variates.

Table 6 shows standardized canonical variate coefficients, correlations between the variates and canonical variates, proportion of variance, redundancy and canonical correlations for the discharge data. Similar to admission, the first pair of canonical variates were moderately related, and the second pair of canonical variates were weakly related. As compared to admission, both pairs of canonical correlates were more correlated in the discharge set.

**Table 6:** Canonical correlation analysis of the discharge dataset. Variables grouped by negative affect/ poor adjustment (Pain Intensity, Depression, Catastrophizing, Anxiety, Somatic Symptoms) and positive affect/ improved adjustment (Activity Engagement, Pain Willingness, Pre-contemplation, Contemplation, Action, Maintenance).

Discharge	First Canonical Variate		Second Canonical Variate	
	Correlation	Function Coefficient	Correlation	Function Coefficient
Negative Affect/ Poor Adjustment Set Variables				
Pain Intensity	0.45	0.10	−0.41	−0.39
Depression	0.79	0.26	−0.52	−0.95
Catastrophizing	0.97	0.79	0.22	1.14
Anxiety	0.70	−0.07	−0.30	−0.11
Somatic Symptoms	0.62	0.04	−0.36	−0.17
Proportion of Variance	0.53		0.14	Total = 0.67
Redundancy	0.31		0.01	Total = 0.32
Positive Affect/ Improved Adjustment Set Variables				
Activity Engagement	−0.78	−0.42	0.41	0.43
Pain Willingness	−0.69	−0.44	−0.52	−0.54
Pre-contemplation	0.76	0.34	0.12	0.57
Contemplation	−0.30	0.07	0.30	−0.12
Action	−0.57	−0.11	0.48	0.20
Maintenance	−0.58	−0.11	0.61	0.68
Proportion of Variance	0.40		0.19	Total = 0.59
Redundancy	0.23		0.02	Total = 0.25
Canonical correlation	0.76		0.30	
Canonical correlation squared	0.58		0.09	

Pain Intensity = Pain Intensity Scale; Depression = Center for Epidemiological Studies-Depressed Mood Scale; Catastrophizing = Pain Catastrophizing Scale; Anxiety = Clinical Anxiety Scale; Somatic Symptoms = Patient Questionnaire of the PRIME-MD; Activity Engagement = Chronic Pain Acceptance Questionnaire – Activity Engagement; Pain Willingness = Chronic Pain Acceptance Questionnaire – Pain Willingness; Pre-contemplation = Pain Stages of Change Questionnaire – Pre-contemplation; Contemplation = Pain Stages of Change Questionnaire – Contemplation; Action = Pain Stages of Change Questionnaire – Action; Maintenance = Pain Stages of Change Questionnaire – Maintenance.

All variables in the negative affect/poor adjustment set were correlated with the first canonical variate, with all correlations of these variables above 0.3 (28). The strongest correlations were again catastrophizing (0.97) and depression (0.79). All variables in the positive affect/ improved adjustment set were correlated with the first canonical variable. The strongest correlation was activity engagement (−0.78) and pre-contemplation (0.76). The first canonical variate indicated that increase in catastrophizing, depression, pain intensity, anxiety, and somatic symptoms was associated with decrease in activity engagement, pain willingness, contemplation, action, maintenance and increase in pre-contemplation.

The second canonical variate consisted of pain intensity (−0.41), depression (−0.52), and somatic symptoms (−0.36) in the negative affect/poor adjustment set, and pain willingness (−0.52), activity engagement (0.41) action (0.48), and maintenance (0.61) in the positive affect/improved adjustment set. The second canonical variate showed that decreased pain intensity, depression and somatic symptoms were associated with increase in activity engagement, action and maintenance and decrease in pain willingness.

Overall, negative affect/poor adjustment is negatively correlated with the positive affect/improved adjustment, with pre-contemplation positively correlated with positive affect/improved adjustment.

## Discussion

The aim of this study was to examine the structure of variables in patients attending a chronic pain program, specifically groupings illustrating positive and negative variables. In the biplot, the overall structure of the variables makes up roughly two groupings. One grouping consists of anxiety, depression, catastrophizing, somatic symptoms, pain intensity and pre-contemplation. The second grouping roughly consists of contemplation, action, maintenance, activity engagement and pain willingness. Pain willingness appears to be the least correlated with the other factors in the second group. These two groupings appear to resonate with the bivalent Behavioral Inhibition System-Behavioral Activation System (BIS-BAS) model (45, 46), with one grouping consisting of generally positive variables and the other representing negative variables. The negative variables appear to consist of anxiety, depression and cognitive content; and the positive variables appear to consist of positive emotions and affect (46). The BIS-BAS model, as proposed by Jensen et al. (2016, 2017) was devised to explain the benefits of psychosocial treatments for chronic pain (45, 46). It hypothesizes the existence of two groups of negative and positive cognitions, affect and behavioral intensions or motivations.

When comparing across admission and discharge biplots, the two groupings of variables become more tightly related at discharge. For example, in the positive variables grouping, contemplation moves closer to activity engagement, action, and maintenance cluster at discharge. This type of change has been previously described in the Dynamic Model of Affect (26) according to which, in stressful situations, the separation between positive and negative emotions decreases. The general pattern of clustering changes between admission and discharge as visualized in the biplots seems to reflect similar notions as those described in the Dynamic Model of Affect. That is, under stressful situations, the emotions become less differentiated.

This mixed state is shown in the admission biplot, with a wide spread of variables whereas in the discharge biplot, the separation between the groupings increases to create more distinct groupings, referring to a change to a possible lower stress state overall at discharge. This in fact, corresponds to the fact that all variables consistently improve at discharge from the four-week interdisciplinary pain management program (47).

Factors in the negative group, such as catastrophizing and anxiety, can be broadly considered to be related to poor psychological adjustment to pain (11). The physical functioning factors, namely pain intensity and somatic symptoms are very closely associated with emotional factors. Thus, physical sensation can be seen as an aspect of emotion. This highlights the importance of psychological treatment (48, 49). With the high correlation of pain intensity and somatic symptoms with these psychological factors, the first grouping may be considered as an aggregation of factors associated with poor psychological adjustment and physical functioning. Catastrophizing, emotional distress, and pain intensity have been found to be closely associated with each other (9, 50, 51). The pre-contemplation subscale of the PSOCQ is also positively associated with the close interplay of negative emotions, cognition, and physical pain. Patients in the pre-contemplation stage are focused on their physical pain and on seeking biomedical solutions (52). This grouping reflects earlier similar findings (23, 53).

In the canonical correlation analysis, at both admission and discharge, all five factors (pain intensity, depression, catastrophizing, anxiety, somatic symptoms) contribute to the poor adjustment canonical variate. Depression, and especially, catastrophizing contributes the most to predicting improved adjustment factors (activity engagement, pain willingness, pre-contemplation, contemplation, action, maintenance) as compared to the physically-focused factors, pain intensity and somatic symptoms.

Catastrophizing is the most influential factor in predicting improved adjustment. It is different from anxiety and depression. In the biplot, catastrophizing was more related to pre-contemplation as compared to other affective factors (depression, anxiety). In patients with chronic pain, depression and anxiety are more closely related to each other as compared with catastrophizing (54). Conceptually, catastrophizing is not pain intensity (55) but catastrophizing may be a mediator within the affective and physical factors such as the indirect effect of pain intensity on depressive symptoms via catastrophizing (7), or as a mediator for depressed mood, pain interference, and pain severity (10, 51, 56). Within the context of the pain experience and its effect on patients’ lives, from the fear-avoidance model, catastrophizing may be a gatekeeper between recovery and the loop of negative affect (54, 57).

In this study, catastrophizing was measured as a single dimension, which limits its interpretability in terms of its three sub-dimensions of rumination, magnification, and helplessness (58). However, it appears that the most notable sub-dimension of catastrophizing is helplessness (58). Helplessness in catastrophizing is related to pain intensity (59). There may be an interplay between wanting to engage in recovery and return to normal living but helplessness keeps patients with chronic pain from doing so in the short term. Depression and anxiety may be related to long-term helplessness, as catastrophizing and depression are risk factors for physical disability and other poor adjustment outcomes in patients with chronic pain (50, 60). Thus, in this canonical correlation analysis specifically, it may be helplessness that most strongly correlates with factors of improved adjustment and functioning. Further analysis may examine this potential relationship.

In both admission and discharge biplots, there is another group of factors that appears generally negatively correlated with factors in the poor adjustment/functioning as mentioned previously. These factors consist of pain willingness, activity, engagement, contemplation, action, and maintenance. These are subdivisions of the chronic pain acceptance questionnaire and the stages of change questionnaire (13, 52), and psychological factors related to improved adjustment to chronic pain (11). The correlations within these factors are weaker as compared to poor adjustment/functioning. The weaker correlation is most noticeable with pain willingness, as it appears to be almost unrelated to both poor and improved adjustment and functioning. In the biplots comparing principal component 1 (PC1) and principal component 2 (PC2), the two adjustment/functioning groupings point in another direction away from pain willingness. This open space may be representative of factors related to positive psychology, such as life satisfaction or self-efficacy. Pain willingness contributes less to the canonical variate as compared to the other subscale of the CPAQ, activities engagement. Pain willingness has been shown to be different from activities engagement, as activities engagement appears to be more closely related to pain intensity and depression in comparison (12, 61–63). Even though it appears to be different from activities engagement in the biplot, pain willingness contributes to the improved adjustment measures in a similar manner as activities engagement.

Further examination is needed to see how other factors fit into this space captured by the biplot. The action and maintenance subscales of the PSOCQ are closely related, and may measure similar concepts (52, 53). Patients with chronic pain were more willing to engage in self-management at discharge (23). In the biplot, at discharge, contemplation becomes more correlated with action and maintenance as compared to admission (64).

Of the six different factors that make up the improved adjustment/functioning canonical variate (pain willingness, activity engagement, pre-contemplation, contemplation, action, and maintenance), activity engagement has the largest contribution to the canonical variate in relating to lower scores on the poor adjustment/functioning measures. High scores on activity engagement, pain willingness and lower scores on pre-contemplation are predictive of lower scores on poor adjustment/functioning. The negative contribution of pre-contemplation corroborates with the pre-contemplation vector placement on the biplot, grouping together with poor adjustment variables. Pre-contemplation is negatively correlated with the rest of the PSOCQ subscales, and has been shown to be correlated with negative pain control, depression, disability and pain severity (23, 53). The contemplation subscale contributed the least to the canonical variate. It has been shown to be different from the other subscales as well (23). At discharge, the contemplation variable became more closely related to the poor adjustment latent. Even though the PSOCQ was developed for assessing patient readiness to engage in self-management at admission to a program or therapy (52), it may be important to re-examine subscales, especially the pre-contemplation and contemplation subscales at discharge as well as when patients move into long-term self-management.

In terms of relating poor adjustment/functioning and improved adjustment/functioning, the most notable relationship between the two groups is highlighted by catastrophizing and activity engagement. This relationship had been conceptualized with the Coping Strategies Questionnaire (CSQ), as catastrophizing in the CSQ was associated with pain interference in activities and increased pain behavior (65, 66). As well, acceptance may be an important factor of consideration for examining catastrophizing (16). It is unclear if pain level is correlated with pain willingness or activity engagement (9). Thus, it may be possible that change in pain cognition, such as catastrophizing, is a more immediate essential treatment outcome as compared to pain willingness, which may reflect behaviors that follow successful cognitive transition to self-management. Further studies may examine this relationship and possible precedents.

In conclusion, this study mapped the overall structure and pattern of interplay between variables in pain management. The relationships were found to be congruent with theories and models of pain psychology. Further work is needed develop these variable structures and improve understanding of treatment outcomes.

Potential limitations include the sample studied. This study utilizes one group of patients from one pain clinic in Canada. However, the length of time in the collected data and the different patient referral sources provide variation in the sample. Another potential limitation is the two time points in the collected data. Additional time points may provide further insight into changes in psychosocial variables. Future studies may examine changes over longer and multiple time points.

## Data Availability

The data analyzed in this study are subject to the following licenses/restrictions: Hospital owned patient information. Requests to access these datasets should be directed to EH, hapidou@hhsc.ca.

## References

[1] Gatchel RJ Peng YB Peters ML Fuchs PN Turk DC. The biopsychosocial approach to chronic pain: scientific advances and future directions. Psychol Bull. (2007) 133:581. 10.1037/0033-2909.133.4.581 17592957

[2] Wootton RJ Warfield CA Bajwa ZH. Principles and practice of pain medicine. 3rd Ed. United States: McGraw-Hill Education (2016). Available at: https://books.google.ca/books?id=6NcUrgEACAAJ

[3] Kroenke K Outcalt S Krebs E Bair MJ Wu J Chumbler N Association between anxiety, health-related quality of life and functional impairment in primary care patients with chronic pain. Gen Hosp Psychiatry. (2013) 35:359–65. 10.1016/j.genhosppsych.2013.03.020 23639186

[4] McWilliams LA Cox BJ Enns MW. Mood and anxiety disorders associated with chronic pain: an examination in a nationally representative sample. Pain. (2003) 106:127–33. 10.1016/S0304-3959(03)00301-4 14581119

[5] Bair MJ Robinson RL Katon W Kroenke K. Depression and pain comorbidity: a literature review. Arch Intern Med. (2003) 163:2433–45. 10.1001/archinte.163.20.2433 14609780

[6] de Heer EW Gerrits MM Beekman AT Dekker J Van Marwijk HW De Waal MW The association of depression and anxiety with pain: a study from NESDA. PLoS One. (2014) 9:e106907. 10.1371/journal.pone.0106907 25330004 PMC4198088

[7] Cheng S-T Leung CM Chan KL Chen PP Chow YF Chung JW The relationship of self-efficacy to catastrophizing and depressive symptoms in community-dwelling older adults with chronic pain: a moderated mediation model. PLoS One. (2018) 13:e0203964. 10.1371/journal.pone.0203964 30226892 PMC6143242

[8] Hülsebusch J Hasenbring MI Rusu AC. Understanding pain and depression in back pain: the role of catastrophizing, help-/hopelessness, and thought suppression as potential mediators. Int J Behav Med. (2016) 23:251–9. 10.1007/s12529-015-9522-y 26590138

[9] Lami MJ Martínez MP Miró E Sánchez AI Guzmán MA. Catastrophizing, acceptance, and coping as mediators between pain and emotional distress and disability in fibromyalgia. J Clin Psychol Med Settings. (2018) 25:80–92. 10.1007/s10880-018-9543-1 29450798

[10] Dong H-J Gerdle B Bernfort L Levin L-Å Dragioti E. Pain catastrophizing in older adults with chronic pain: the mediator effect of mood using a path analysis approach. J Clin Med. (2020) 9:2073. 10.3390/jcm9072073 32630330 PMC7408783

[11] Keefe FJ Rumble ME Scipio CD Giordano LA Perri LM. Psychological aspects of persistent pain: current state of the science. J Pain. (2004) 5:195–211. 10.1016/j.jpain.2004.02.576 15162342

[12] Carvalho SA Gillanders D Palmeira L Pinto-Gouveia J Castilho P. Mindfulness, selfcompassion, and depressive symptoms in chronic pain: the role of pain acceptance. J Clin Psychol. (2018) 74:2094–106. 10.1002/jclp.22689 30101973

[13] McCracken LM Vowles KE Eccleston C. Acceptance of chronic pain: component analysis and a revised assessment method. Pain. (2004) 107:159–66. 10.1016/j.pain.2003.10.012 14715402

[14] Ravn SL Vang ML Vaegter HB Andersen TE. Pain-related acceptance as a mediator in the fear avoidance model of chronic pain: a preliminary study. Pain Medicine. (2018) 19:1764–71. 10.1093/pm/pnx223 29036699

[15] Miró J Castarlenas E de la Vega R Galán S Sanchez-Rodriguez E Jensen MP Pain catastrophizing, activity engagement and pain willingness as predictors of the benefits of multidisciplinary cognitive behaviorally-based chronic pain treatment. J Behav Med. (2018) 41:827–35. 10.1007/s10865-018-9927-6 29736780

[16] Esteve R Ramírez-Maestre C López-Martínez AE. Adjustment to chronic pain: the role of pain acceptance, coping strategies, and pain-related cognitions. Ann Behav Med. (2007) 33:179–88. 10.1007/BF02879899 17447870

[17] Kerns RD Wagner J Rosenberg R Haythornthwaite J Caudill-Slosberg M. Identification of subgroups of persons with chronic pain based on profiles on the pain stages of change questionnaire. Pain. (2005) 116:302–10. 10.1016/j.pain.2005.04.022 15985332

[18] Krebs P Norcross JC Nicholson JM Prochaska JO. Stages of change and psychotherapy outcomes: a review and meta-analysis. J Clin Psychol. (2018) 74:1964–79. 10.1002/jclp.22683 30335193

[19] Hadjistavropoulos H Shymkiw J. Predicting readiness to self-manage pain. Clin J Pain. (2007) 23:259–66. 10.1097/AJP.0b013e31802f67f3 17314586

[20] Jensen MP Nielson WR Turner JA Romano JM Hill ML. Changes in readiness to self-manage pain are associated with improvement in multidisciplinary pain treatment and pain coping. Pain. (2004) 111:84–95. 10.1016/j.pain.2004.06.003 15327812

[21] Williams RM Hapidou EG Lin C-YA Abbasi H. Examining the pain stages of change questionnaire in chronic pain. Physiotherapy Canada. (2007) 59:132–41. 10.3138/ptc.59.2.132

[22] Fletcher R Braithwaite FA Woodhouse M MacInnes A Stanton TR. Does readiness to change influence pain-related outcomes after an educational intervention for people with chronic pain? A pragmatic, preliminary study. Physiother Theory Pract. (2021) 37:608–19. 10.1080/09593985.2019.1636436 31267821

[23] Jensen MP Nielson WR Turner JA Romano JM Hill ML. Readiness to self-manage pain is associated with coping and with psychological and physical functioning among patients with chronic pain. Pain. (2003) 104:529–37. 10.1016/S0304-3959(03)00092-7 12927625

[24] Strand EB Kerns RD Christie A Haavik-Nilsen K Klokkerud M Finset A. Higher levels of pain readiness to change and more positive affect reduce pain reports–a weekly assessment study on arthritis patients. Pain. (2007) 127:204–13. 10.1016/j.pain.2006.08.015 16997472

[25] Finan PH Garland EL. The role of positive affect in pain and its treatment. Clin J Pain. (2015) 31(2):177–87. 10.1097/AJP.0000000000000092 24751543 PMC4201897

[26] Zautra A Smith B Affleck G Tennen H. Examinations of chronic pain and affect relationships: applications of a dynamic model of affect. J Consult Clin Psychol. (2001) 69:786. 10.1037/0022-006X.69.5.786 11680555

[27] Li Y Hapidou EG. Patient satisfaction with chronic pain management: patient perspectives of improvement. Journal of Patient Experience. (2021) 8:23743735211007830. 10.1177/23743735211007834 PMC8205408 34179424

[28] Tabachnick BG Fidell LS Ullman JB. Using multivariate statistics. Boston: Pearson (2019).

[29] Husson F Lê S Pagès J. Exploratory multivariate analysis by example using R. New York: Taylor & Francis Limited (2020). Available at: https://books.google.ca/books?id=xyu5zQEACAAJ

[30] Everitt B Hothorn T. An introduction to applied multivariate analysis with R. New York: Springer Science & Business Media (2011). 284.

[31] Gabriel KR. The biplot graphic display of matrices with application to principal component analysis. Biometrika. (1971) 58:453–67. 10.1093/biomet/58.3.453

[32] Jacoby WG. Statistical graphics for visualizing multivariate data. Thousand Oaks: Sage Publications (1998). p. 120. Available at: http://archive.org/details/statisticalgraph0000jaco (Accessed March 28, 2022).

[33] Reading and understanding MORE multivariate statistics. Washington, DC, US: American Psychological Association (2000). p. 437.

[34] Sherry A Henson RK. Conducting and interpreting canonical correlation analysis in personality research: a user-friendly primer. J Pers Assess. (2005) 84:37–48. 10.1207/s15327752jpa8401_09 15639766

[35] Dunteman GH. Principal components analysis. California: SAGE Publications (1989). Available at: https://books.google.ca/books?id=Pzwt-CMMt4UC

[36] R Core Team. R: A language and environment for statistical computing. Vienna, Austria: R Foundation for Statistical Computing (2021). Available at: https://www.R-project.org/

[37] RStudio Team. RStudio: integrated development environment for R. Boston, MA (2021). Available at: http://www.rstudio.com/

[38] Sievert C. Interactive web-based data visualization with r, plotly, and shiny. Florida: Chapman and Hall/CRC (2020). Available at: https://plotly-r.com

[39] Revelle W. Psych: procedures for psychological, psychometric, and personality research. Evanston, Illinois (2021). Available at: https://CRAN.R-project.org/package=psych

[40] González I Déjean S. CCA: canonical correlation analysis. (2021). Available at: https://CRAN.R-project.org/package=CCA

[41] Menzel U. CCP: significance tests for canonical correlation analysis (CCA). (2012). Available at: https://CRAN.R-project.org/package=CCP

[42] Dziuban CD Shirkey EC. When is a correlation matrix appropriate for factor analysis? Some decision rules. Psychol Bull. (1974) 81:358. 10.1037/h0036316

[43] Schumacker RE. Using R with multivariate statistics. 1st edition. California: SAGE Publications, Inc (2015). 406 p.

[44] Meyers LS Gamst G Guarino AJ. Applied multivariate research: design and interpretation. California: Sage publications (2016).

[45] Jensen MP Ehde DM Day MA. The behavioral activation and inhibition systems: implications for understanding and treating chronic pain. J Pain. (2016) 17:529–e1. 10.1016/j.jpain.2016.02.001 27052644

[46] Jensen MP Ward LC Thorn BE Ehde DM Day MA. Measuring the cognitions, emotions, and motivation associated with avoidance behaviors in the context of pain. Clin J Pain. (2017) 33:325–34. 10.1097/AJP.0000000000000407 27428549

[47] Hapidou EG Pham E Bartley K Anthonypillai J Altena S Patterson L Chronic pain program management outcomes: long-term follow-up for veterans and civilians. J Mil Veteran Fam Health. (2021) 7:74–91. 10.3138/jmvfh-2021-0054

[48] Romano JM Turner JA. Chronic pain and depression: does the evidence support a relationship? Psychol Bull. (1985) 97(1):18–34. 10.1037/0033-2909.97.1.18 3983297

[49] Scascighini L Toma V Dober-Spielmann S Sprott H. Multidisciplinary treatment for chronic pain: a systematic review of interventions and outcomes. Rheumatology. (2008) 47:670–8. 10.1093/rheumatology/ken021 18375406

[50] Edwards RR Giles J Bingham CO III Campbell C Haythornthwaite JA Bathon J. Moderators of the negative effects of catastrophizing in arthritis. Pain Medicine. (2010) 11:591–9. 10.1111/j.1526-4637.2010.00804.x 20210869 PMC2868122

[51] Martinez-Calderon J Jensen MP Morales-Asencio JM Luque-Suarez A. Pain catastrophizing and function in individuals with chronic musculoskeletal pain. Clin J Pain. (2019) 35:279–93. 10.1097/AJP.0000000000000676 30664551

[52] Kerns RD Rosenberg R Jamison RN Caudill MA Haythornthwaite J. Readiness to adopt a self-management approach to chronic pain: the pain stages of change questionnaire (PSOCQ). PAIN. (1997) 72:227–34. 10.1016/S0304-3959(97)00038-9 9272807

[53] Jensen MP Nielson WR Romano JM Hill ML Turner JA. Further evaluation of the pain stages of change questionnaire: is the transtheoretical model of change useful for patients with chronic pain? Pain. (2000) 86:255–64. 10.1016/S0304-3959(00)00257-8 10812255

[54] Turk DC Fillingim RB Ohrbach R Patel KV. Assessment of psychosocial and functional impact of chronic pain. J Pain. (2016) 17:T21–49. 10.1016/j.jpain.2016.02.006 27586830

[55] Furrer A Michel G Terrill AL Jensen MP Müller R. Modeling subjective well-being in individuals with chronic pain and a physical disability: the role of pain control and pain catastrophizing. Disabil Rehabil. (2019) 41:498–507. 10.1080/09638288.2017.1390614 29057668

[56] Craner JR Lake ES Bancroft KA George LL. Treatment outcomes and mechanisms for an ACT-based 10-week interdisciplinary chronic pain rehabilitation program. Pain Pract. (2020) 20:44–54. 10.1111/papr.12824 31336019

[57] Vlaeyen JW Linton SJ. Fear-avoidance and its consequences in chronic musculoskeletal pain: a state of the art. Pain. (2000) 85:317–32. 10.1016/S0304-3959(99)00242-0 10781906

[58] Gilliam WP Craner JR Morrison EJ Sperry JA. The mediating effects of the different dimensions of pain catastrophizing on outcomes in an interdisciplinary pain rehabilitation program. Clin J Pain. (2017) 33:443–51. 10.1097/AJP.0000000000000419 27437567

[59] Adachi T Nakae A Maruo T Shi K Maeda L Saitoh Y The relationships between pain-catastrophizing subcomponents and multiple pain-related outcomes in Japanese outpatients with chronic pain: a cross-sectional study. Pain Pract. (2019) 19:27–36. 10.1111/papr.12712 29772106

[60] Lazaridou A Martel MO Cornelius M Franceschelli O Campbell C Smith M The association between daily physical activity and pain among patients with knee osteoarthritis: the moderating role of pain catastrophizing. Pain Medicine. (2019) 20:916–24. 10.1093/pm/pny129 30016486 PMC6497093

[61] Day MA Thorn BE. The mediating role of pain acceptance during mindfulness-based cognitive therapy for headache. Complement Ther Med. (2016) 25:51–4. 10.1016/j.ctim.2016.01.002 27062948

[62] Nicholas MK Asghari A. Investigating acceptance in adjustment to chronic pain: is acceptance broader than we thought? Pain. (2006) 124:269–79. 10.1016/j.pain.2006.04.032 16934925

[63] Sánchez-Rodríguez E Racine M Castarlenas E Tomé-Pires C Galán S Jensen MP Behavioral activation and inhibition systems: further evaluation of a BIS-BAS model of chronic pain. Pain Medicine. (2021) 22:848–60. 10.1093/pm/pnaa330 33249468

[64] Kerns RD Rosenberg R. Predicting responses to self-management treatments for chronic pain: application of the pain stages of change model. Pain. (2000) 84:49–55. 10.1016/S0304-3959(99)00184-0 10601672

[65] Sullivan MJ Thorn B Haythornthwaite JA Keefe F Martin M Bradley LA Theoretical perspectives on the relation between catastrophizing and pain. Clin J Pain. (2001) 17:52–64. 10.1097/00002508-200103000-00008 11289089

[66] Turner JA Jensen MP Warms CA Cardenas DD. Catastrophizing is associated with pain intensity, psychological distress, and pain-related disability among individuals with chronic pain after spinal cord injury. Pain. (2002) 98:127–34. 10.1016/S0304-3959(02)00045-3 12098624

